# Public Awareness, Perceptions, and Treatment Preferences Regarding Metabolic (Bariatric) Surgery and Injectable Pharmacotherapies for Type 2 Diabetes in Jeddah, Saudi Arabia: A Cross-Sectional Analysis

**DOI:** 10.7759/cureus.103354

**Published:** 2026-02-10

**Authors:** Yusef H Albog, Ahmed Elshora, Ahmed I ALzaydani, Sami M Jameel, Khaled M Albasheiti, Amira M Almaqri, Halah D Alshehri, Nuha Fatima, Mesheal Alanazi, Suad M Alkhalaf, Yousef J Albalawi, Rama H Albooq

**Affiliations:** 1 Medicine and Surgery, Batterjee Medical College, Jeddah, SAU; 2 General Surgery, Batterjee Medical College, Jeddah, SAU; 3 Internal Medicine, Batterjee Medical College, Jeddah, SAU; 4 Surgery, Saudi German Hospital, Jeddah, SAU; 5 Surgery, Batterjee Medical College, Jeddah, SAU; 6 General Medicine, Batterjee Medical College, Jeddah, SAU

**Keywords:** glp-1 agonists, metabolic (bariatric) surgery, public awareness, saudi arabia, treatment preference, type 2 diabetes

## Abstract

Background

Obesity and type 2 diabetes mellitus (T2DM) represent major and rapidly increasing public health burdens in Saudi Arabia. Metabolic (bariatric) surgery is an effective long-term intervention for obesity-related T2DM, while modern injectable pharmacotherapies, including glucagon-like peptide-1 receptor agonists, have become increasingly popular. However, public awareness and perceptions regarding these modalities remain insufficiently characterized in Saudi communities. Hence, this study aimed to assess public awareness, perceptions, misconceptions, and self-reported treatment preferences regarding metabolic (bariatric) surgery and injectable pharmacotherapies for obesity-related T2DM among adult residents of Jeddah, Saudi Arabia, using a perception-based cross-sectional survey.

Methodology

A community-based, descriptive, cross-sectional survey was conducted using an online, self-administered questionnaire distributed via social media platforms. The survey assessed sociodemographic characteristics, self-reported diabetes status, awareness and perceptions of obesity, metabolic (bariatric) surgery, and injectable pharmacotherapies, as well as stated treatment preferences. All outcomes reflect participant perceptions and were not intended to measure clinical knowledge or treatment effectiveness. Accordingly, type 2 diabetes status and related variables were self-reported and not verified through medical records. Descriptive statistics were used to summarize participant characteristics and awareness indicators. Chi-square tests were applied to explore exploratory associations between selected demographic variables and treatment preferences, with statistical significance set at a p-value <0.05.

Results

Participants demonstrated high awareness that obesity is a major modifiable risk factor for T2DM (74.6%). However, only 21.1% of participants recognized metabolic (bariatric) surgery as an effective metabolic intervention for diabetes improvement. Injectable therapies were preferred by 57.9% of participants, while 42.1% preferred surgical options. Misconceptions regarding indications, risks, and long-term outcomes were prevalent.

Conclusions

Although general awareness of obesity’s metabolic impact was high, understanding of the therapeutic role of metabolic (bariatric) surgery for T2DM remained limited. Injectable therapies were more frequently preferred than surgical options; however, the present data do not allow definitive conclusions regarding the underlying drivers of these preferences. These findings highlight the need for structured public education initiatives to address misconceptions and improve metabolic literacy. The results reflect public perceptions rather than clinical decision-making or treatment effectiveness.

## Introduction

Obesity has emerged as one of the most pressing global health concerns, contributing substantially to the development and progression of type 2 diabetes mellitus (T2DM), cardiovascular disease, and a broad spectrum of metabolic complications [1-5]. In Saudi Arabia, the burden of obesity has reached critical levels, with national prevalence estimates ranking among the highest worldwide [6-10]. This escalating burden has translated into considerable clinical, economic, and social consequences, placing sustained pressure on primary care services, long-term chronic disease management, and specialized endocrinology clinics [2,5,10].

A strong biological relationship links excess adiposity to insulin resistance and progressive metabolic dysfunction. Adipose tissue expansion and dysfunction can promote chronic low-grade inflammation, altered adipokine signaling, and impaired glucose homeostasis, thereby accelerating the onset and worsening of T2DM [3-5,11,12]. Consequently, early recognition and effective management of obesity are increasingly viewed as central pillars for diabetes prevention and improved metabolic control [4,5].

Metabolic (bariatric) surgery has consistently demonstrated superiority over conventional medical therapy in achieving sustained weight reduction, improving glycemic control, and reducing long-term diabetes complications [13-18]. Procedures such as laparoscopic sleeve gastrectomy and Roux-en-Y gastric bypass have been associated with meaningful improvements in β-cell function, insulin sensitivity, lipid profiles, and cardiovascular risk markers [13-20]. International guidelines and expert consensus statements endorse metabolic surgery as a key therapeutic option for individuals with T2DM and obesity who fail to achieve adequate control with lifestyle modification and pharmacotherapy [21-26].

Despite robust evidence supporting its clinical effectiveness, metabolic (bariatric) surgery remains underutilized, and public perceptions appear to influence treatment uptake [27-29]. Misconceptions regarding surgical safety, long-term outcomes, candidacy criteria, and metabolic benefits, along with cultural beliefs, fear of complications, and social stigma, may shape decision-making [27-29]. In Saudi Arabia, where obesity and diabetes prevalence are high [6-10], understanding public beliefs is essential for guiding health policy, patient education, and clinical counseling strategies [6-10,27,28].

In parallel with surgical advances, pharmacological management of obesity and T2DM has evolved substantially. Injectable agents, particularly glucagon-like peptide-1 (GLP-1) receptor agonists and newer dual incretin therapies, have gained broad attention for their effects on weight loss and glycemic improvement [30-33]. This growing visibility may shift preferences toward less invasive modalities; however, real-world understanding of mechanisms, limitations, and long-term outcomes remains variable [30-33].

Given these trends, assessing public awareness and preferences regarding both metabolic (bariatric) surgery and injectable therapies is increasingly important [21-33]. To date, relatively few studies in Saudi Arabia have evaluated perceptions of both modalities simultaneously in large samples [6-10,27,28]. Therefore, this study aimed to provide an in-depth analysis of public awareness, perceptions, and treatment preferences related to metabolic (bariatric) surgery and injectable therapies for T2DM among adults in Saudi Arabia using a large cross-sectional survey. This study was intentionally designed as a descriptive, perception-based investigation. It does not aim to evaluate clinical outcomes, treatment efficacy, or guideline adherence, but rather to explore community-level awareness and beliefs regarding metabolic treatment options.

## Materials and methods

Survey distribution

The survey was distributed online via social media platforms (e.g., WhatsApp and X) and was open between May 6, 2025, and August 29, 2025. Because the survey link was disseminated publicly through multiple channels, a response rate could not be calculated. Because this was a convenience-based online survey, the study was not intended to generate population-representative estimates for Jeddah. Findings reflect the perceptions of surveyed participants rather than the entire population of the city.

Study measures

The survey instrument was designed to capture comprehensive information across multiple domains, including demographic characteristics, obesity- and diabetes-related knowledge, awareness of metabolic (bariatric) surgery, and preferences toward injectable pharmacotherapies. Demographic variables included age, gender, nationality, marital status, and educational level, as these factors have been shown to influence health literacy, diabetes awareness, and treatment decision-making in Middle Eastern populations [1-4].

Anthropometric data were collected using self-reported weight and height and were used to calculate body mass index (BMI). Participants were categorized according to standard World Health Organization (WHO) BMI classifications (normal weight, overweight, and obesity classes I-III) [9]. This classification framework is publicly available and free to use. BMI categorization enabled assessment of how individual weight status may influence perceptions and preferences toward metabolic (bariatric) surgery or pharmacological therapy.

Diabetes-related variables included self-reported type 2 diabetes status, presence of diabetes-related complications (neuropathy, retinopathy, nephropathy, cardiovascular disease), number of antidiabetic medications used daily, and coexisting chronic conditions such as hypertension, gastroesophageal reflux disease, asthma, and obstructive sleep apnea [11,17]. Type 2 diabetes status and complications were self-reported and were not verified through medical records or laboratory testing.

Awareness of obesity and its metabolic impact was assessed by evaluating participants’ understanding of obesity as a modifiable risk factor for type 2 diabetes, awareness of the relationship between weight loss and glycemic improvement, and knowledge of obesity-related complications, consistent with previously published frameworks [5,10,11]. Responses were captured using agreement-based items to assess general metabolic literacy.

Awareness of metabolic (bariatric) surgery was evaluated by assessing participants’ familiarity with bariatric procedures (sleeve gastrectomy, gastric bypass, mini-gastric bypass, and gastric banding) and their perceptions regarding the metabolic benefits of surgery, including improvements in insulin sensitivity, reduction in medication burden, and prevention of long-term complications [2,4,7,18,27].

Awareness of injectable pharmacotherapy focused on familiarity with anti-obesity and GLP-1 receptor agonist-based injectable medications, perceived effectiveness, and comparison with surgical treatment options, consistent with contemporary diabetes and obesity management literature [21-24].

Treatment preferences were assessed by comparing participants’ willingness to undergo metabolic (bariatric) surgery versus injectable therapy. Participants selected their preferred treatment modality and provided reasons underlying their choice, allowing exploration of cultural, psychological, and informational factors influencing treatment decision-making.

The questionnaire was developed by the authors based on previously published literature and consisted exclusively of non-proprietary, self-constructed items tailored to the study objectives and culturally adapted for the Saudi population [1-4,21-24,27-29,32-35]. No copyrighted, licensed, or proprietary scales, scoring systems, or instruments were used. Therefore, no permissions or license agreements were required. In addition, no figures or tables were reproduced or adapted from copyrighted sources; all tables were generated directly from the study dataset.

Pilot testing

Before distribution, the questionnaire underwent pilot testing among 20 individuals to ensure clarity, readability, and cultural appropriateness. Minor adjustments to wording and response options were made based on pilot feedback.

Reliability and validity

Internal consistency of multi-item constructs (e.g., obesity awareness, bariatric benefits, treatment preference) was evaluated using Cronbach’s alpha during the pilot phase. All assessed domains achieved acceptable reliability thresholds (α ≥ 0.72), indicating strong internal coherence of the questionnaire sections. Content validity was supported through literature-based item development and pilot testing. While formal external validation was not performed, internal consistency analysis demonstrated acceptable reliability for perception-based research.

Statistical analysis

Data analysis was performed using SPSS Statistics, version 26 (IBM Corp., Armonk, NY, USA). Descriptive statistics were used to summarize sociodemographic variables, BMI categories, diabetes characteristics, awareness indicators, and treatment preferences. Categorical variables were expressed as frequencies and percentages. Continuous variables (e.g., age, BMI) were expressed as means ± standard deviation (SD) when applicable. Awareness items and treatment preferences were analyzed using cross-tabulation to explore patterns among demographic subgroups. Where appropriate, chi-square tests were used to evaluate associations between demographic factors (age group, gender, BMI category, educational level) and treatment preferences (metabolic (bariatric) surgery vs. injectable therapy). Age groups were categorized using commonly applied groupings in regional public health surveys to allow comparison across life stages and consistency with prior Saudi research. Significance was set at p-values <0.05 for all analyses. Although multivariable modeling (e.g., logistic regression) can strengthen inference, it was not performed because the survey was primarily designed to describe awareness and preferences and did not capture standardized explanatory drivers for all participants. Therefore, inferential findings are presented as exploratory based on chi-square comparisons.

Study outcomes

The study focused on three main outcome domains. The first domain assessed awareness-related outcomes, including participants’ knowledge of obesity as a modifiable risk factor for type 2 diabetes, awareness of metabolic improvement following metabolic (bariatric) surgery, and familiarity with injectable pharmacological therapies such as GLP-1 receptor agonists. The second domain evaluated clinical burden outcomes, including the prevalence of type 2 diabetes, self-reported diabetes-related complications, and the number of antidiabetic medications used. The third domain examined treatment preference outcomes, specifically participants’ preference for surgical versus non-surgical management strategies and the factors influencing these choices, such as perceived safety, effectiveness, cost, and invasiveness.

## Results

A total of 582 participants completed the survey, providing insight into perceptions among surveyed adult residents of Jeddah. The sociodemographic characteristics of the study population are summarized in Table 1. The majority of respondents were female, and most participants belonged to younger age groups, particularly those aged 18-25 years and 26-35 years. Most participants were Saudi nationals, and educational attainment varied, with bachelor’s degree holders constituting the largest subgroup. Nearly half of the participants reported previous attempts at weight-loss interventions; however, only a minority perceived these attempts as successful.

**Table 1:** Sociodemographic characteristics (n = 582).

Variable	n	%
Gender		
Male	223	38.3
Female	359	61.7
Age groups		
18–25 years	343	58.9
26–35 years	116	19.9
36–45 years	24	4.1
≥46 years	99	17.0
Nationality		
Saudi	541	92.9
Non-Saudi	41	7.1
Marital status		
Single	387	66.5
Married	133	22.8
Divorced	19	3.3
Widowed	43	7.4
Education level		
High school	58	10.0
Diploma	89	15.3
Bachelor’s degree	334	57.4
Master’s/PhD	101	17.3

BMI classification demonstrated a high prevalence of excess weight within the sample. Overweight and obesity were common, with obesity classes I-III accounting for the largest proportion of participants, reflecting the substantial burden of obesity in the community (Table 2).

**Table 2:** BMI classification of participants (n = 582) BMI categories were classified according to WHO guidelines [9]. BMI = body mass index; WHO = World Health Organization

BMI category	n	%
Normal	20	3.4
Overweight	62	10.7
Obesity Class I	147	25.3
Obesity Class II	239	41.1
Obesity Class III	114	19.6

Regarding diabetes status, approximately half of the participants reported having T2DM, while the remainder reported no diabetes diagnosis. This finding should be interpreted cautiously, as diabetes status was self-reported and not verified clinically, which may reflect misunderstanding or misclassification. Diabetes-related characteristics, including awareness of complications and medication use, are presented in Table 3. Among participants with diabetes, only a small proportion reported confirmed complications, whereas the majority selected “I don’t know,” indicating considerable uncertainty regarding their complication status. When asked about specific perceived complications, sensory loss, visual problems, and cardiovascular conditions were most frequently reported, while kidney disease and hearing impairment were less commonly identified. Medication use varied widely, with most participants reporting no diabetes medications, fewer reporting one or two agents, and only a small fraction reporting the use of four or more medications. Several comorbid conditions, including obesity, hypertension, asthma, gastroesophageal reflux disease, and obstructive sleep apnea, were also prevalent, further highlighting the metabolic burden within the cohort.

**Table 3:** Diabetes-related characteristics (n = 582).

Variable	n	%
Type 2 diabetes diagnosis		
Yes	287	49.3
No	295	50.7
Diabetes complications		
Yes	20	3.4
No	266	45.7
I don’t know	296	50.9
Perceived diabetes-related complications (multiple response; overall sample)		
Loss of sensation	488	83.8
Eye problems	329	56.5
Kidney disease	132	22.7
Heart disease	289	49.7
Hearing impairment	80	13.7
Others	55	9.5
Number of diabetes medications		
0	381	65.5
1	103	17.7
2	38	6.5
3	29	5.0
4	18	3.1
>4	13	2.2

Participants demonstrated substantial awareness of obesity and its metabolic implications. Awareness regarding the role of obesity in diabetes, the impact of weight loss on glycemic control, and the association between obesity and diabetes-related complications is summarized in Table 4. Although general metabolic awareness was high, this knowledge did not consistently extend to advanced therapeutic interventions.

**Table 4:** Awareness of obesity, diabetes, and related concepts. T2DM = type 2 diabetes mellitus

Awareness item	Yes (%)	No (%)	I don’t know (%)
Do you know the role of obesity in diabetes?	387 (66.5%)	195 (33.5%)	—
Obesity is the main modifiable risk factor for T2DM	434 (74.6%)	148 (25.4%)	—
Does weight loss improve diabetes?	389 (66.8%)	93 (16.0%)	100 (17.2%)
Does obesity increase the risk of diabetes complications?	453 (77.8%)	129 (22.2%)	—

The history of metabolic (bariatric) surgery among participants is shown in Table 5. Only a minority reported having undergone metabolic (bariatric) surgery. However, familiarity with different surgical procedures was relatively high, with sleeve gastrectomy being the most recognized procedure, followed by gastric bypass, mini-gastric bypass, and gastric banding (Table 6). These findings reflect recognition of procedure names rather than procedures actually undertaken. Despite this familiarity, the willingness to pursue surgical intervention was low, with most participants expressing reluctance or uncertainty. Furthermore, the majority perceived previous weight-loss attempts as unsuccessful, underscoring the chronic and relapsing nature of obesity.

**Table 5:** History of metabolic (bariatric) surgery among participants (n = 582).

Variable	n	%
Underwent metabolic (bariatric) surgery		
Yes	90	15.5
No	492	84.5

**Table 6:** Recognized types of metabolic (bariatric) surgery (multiple response; familiarity only). Percentages may exceed 100% because multiple responses were permitted. Values reflect familiarity with procedure names and do not indicate procedures previously undergone.

Procedure	n	%
Sleeve gastrectomy	419	72.0
Gastric bypass	63	10.8
Mini-gastric bypass	45	7.7
Gastric banding	25	4.3
Others	30	5.2

Awareness of injectable pharmacotherapies for weight loss and glycemic control was comparatively higher. Familiarity with injectable treatments and preferences between surgical and pharmacological options are summarized in Table 7. Most participants reported awareness of injectable therapies, and this awareness translated into a stronger preference for pharmacological treatment over surgical options when considering clinician-recommended interventions.

**Table 7:** Awareness of injectable pharmacological options. T2DM = type 2 diabetes mellitus

Variable	n	%
Awareness of injectables for weight loss and T2DM		
Yes	322	55.3
No	260	44.7
Preference: surgical vs. pharmacological		
Surgical option	245	42.1
Pharmacological option	337	57.9

When participants were asked to identify potential benefits of metabolic (bariatric) surgery, many acknowledged advantages such as improved insulin sensitivity, reduced medication burden, and prevention of long-term complications. Nevertheless, the level of agreement remained lower than that observed for injectable therapies. Overall, these findings indicate substantial heterogeneity in public understanding of metabolic treatment strategies. While general awareness of obesity as a contributor to type 2 diabetes was high, knowledge regarding the metabolic role of bariatric surgery in improving glycemic outcomes remained limited. In contrast, awareness of injectable pharmacotherapies was higher and accompanied by a greater preference for pharmacological approaches. Given the community-based online sampling design, these results should be interpreted as reflecting participant perceptions rather than population-level estimates.

## Discussion

Participants demonstrated strong general awareness of obesity as a modifiable risk factor for type 2 diabetes, and many recognized that weight loss can improve glycemic control, consistent with prior international and regional literature [4-10,30,31]. Awareness that obesity increases the risk of diabetes-related complications was also relatively high, aligning with published Saudi and regional data on metabolic risk perception [6-10].

However, this general understanding did not consistently translate into knowledge of the metabolic benefits of metabolic (bariatric) surgery. Although substantial evidence supports surgery in improving insulin sensitivity, reducing medication burden, and achieving remission in selected patients [13-26], only a smaller proportion of respondents believed that metabolic (bariatric) surgery directly improves diabetes outcomes. Similar gaps in perception, often driven by fear of complications and limited access to accurate information, have been described in Saudi and Gulf communities [27,28].

In contrast, awareness of injectable pharmacotherapy, particularly GLP-1-based and newer incretin therapies, was higher, and this familiarity was accompanied by a stronger preference for pharmacologic treatment compared with surgical options [30-33]. This pattern may reflect the increasing visibility and real-world adoption of injectables, even though long-term comparative effectiveness can differ by patient profile and treatment goals [13-20,32,33]. Notably, while many respondents reported unsuccessful prior weight-loss attempts, preference still leaned toward less invasive approaches, echoing prior observations that perceived invasiveness and safety concerns can outweigh potential long-term metabolic benefits in shaping public choices [13-20,27-29].

When participants were asked about potential benefits of metabolic surgery, many acknowledged advantages such as improved insulin sensitivity, reduced medication requirements, and prevention of long-term complications, outcomes supported by randomized trials, meta-analyses, and long-term cohort evidence [13-20,21-26]. Nevertheless, agreement remained lower than the awareness observed for injectable therapies, suggesting persistent uncertainty about surgical mechanisms and long-term safety. This perception gap has also been reported in multiple settings, where misconceptions and stigma may reduce surgical acceptability despite established metabolic benefits [27-29].

Overall, the findings highlight heterogeneity in public understanding of metabolic treatment strategies. While general awareness of obesity’s relationship to diabetes was relatively strong [4-10], perceived risk-benefit balance remained skewed toward injectables, despite robust evidence supporting durable metabolic outcomes with surgery in appropriately selected patients [13-26,34,35]. These results underscore the need for targeted educational approaches that address misconceptions, clarify eligibility and safety, and explain the complementary roles of surgical and pharmacologic pathways in obesity and diabetes management [21-26,27-29,35]. Observed preferences should not be interpreted as recommendations or indicators of optimal treatment choice, but rather as reflections of current public perceptions shaped by familiarity, accessibility, and perceived invasiveness.

Limitations

This study has limitations typical of perception-based cross-sectional surveys. All data, including type 2 diabetes status and anthropometric measures, were self-reported and not clinically verified, which may have resulted in misclassification. The use of a non-probability, online convenience sample introduces selection bias and limits generalizability; therefore, findings should not be considered representative of the entire population of Jeddah or Saudi Arabia. Although the questionnaire was developed based on published literature, pilot tested, and demonstrated acceptable internal consistency, formal external validation was not performed. In addition, the cross-sectional design and exploratory analyses preclude causal inference. Accordingly, the results reflect public perceptions and self-reported preferences rather than clinical outcomes or treatment effectiveness.

## Conclusions

This large community-based study highlights that while awareness of obesity as a key modifiable risk factor for type 2 diabetes is high in Saudi Arabia, knowledge about the metabolic benefits of bariatric surgery remains limited. Injectable pharmacotherapies, particularly GLP-1-based agents, were more widely recognized and preferred, despite evidence supporting the superior long-term metabolic outcomes of surgical interventions. The findings are intended to inform future public education strategies and hypothesis-generating research rather than guide clinical practice or policy decisions.
